# FAM20C directly binds to and phosphorylates Periostin

**DOI:** 10.1038/s41598-020-74400-6

**Published:** 2020-10-13

**Authors:** Ju-Hsien Lin, I-Ping Lin, Yoshio Ohyama, Hanna Mochida, Akira Kudo, Masaru Kaku, Yoshiyuki Mochida

**Affiliations:** 1 Department of Molecular and Cell Biology, Henry M. Goldman School of Dental Medicine, Boston University, Boston, MA USA; 2 Graduate Institute of Clinical Dentistry, School of Dentistry, College of Medicine, National Taiwan University, Taipei, Taiwan; 3 Department of Biological Information, Graduate School of Bioscience and Biotechnology, Tokyo Institute of Technology, Yokohama, Japan; 4 Division of Bio-Prosthodontics, Niigata University Graduate School of Medical and Dental Sciences, Niigata, Japan

**Keywords:** Post-translational modifications, Post-translational modifications

## Abstract

It is widely accepted that FAM20C functions as a Golgi casein kinase and has large numbers of kinase substrates within the secretory pathway. It has been previously reported that FAM20C is required for maintenance of healthy periodontal tissues. However, there has been no report that any extracellular matrix molecules expressed in periodontal tissues are indeed substrates of FAM20C. In this study, we sought to identify the binding partner(s) of FAM20C. FAM20C wild-type (WT) and its kinase inactive form D478A proteins were generated. These proteins were electrophoresed and the Coomassie Brilliant Blue (CBB)-positive bands were analyzed to identify FAM20C-binding protein(s) by Mass Spectrometry (MS) analysis. Periostin was found by the analysis and the binding between FAM20C and Periostin was investigated in cell cultures and in vitro. We further determined the binding region(s) within Periostin responsible for FAM20C-binding. Immunolocalization of FAM20C and Periostin was examined using mouse periodontium tissues by immunohistochemical analysis. In vitro kinase assay was performed using Periostin and FAM20C proteins to see whether FAM20C phosphorylates Periostin in vitro. We identified Periostin as one of FAM20C-binding proteins by MS analysis. Periostin interacted with FAM20C in a kinase-activity independent manner and the binding was direct in vitro. We further identified the binding domain of FAM20C in Periostin, which was mapped within Fasciclin (Fas) I domain 1–4 of Periostin. Immunolocalization of FAM20C was observed in periodontal ligament (PDL) extracellular matrix where that of Periostin was also immunostained in murine periodontal tissues. FAM20C WT, but not D478A, phosphorylated Periostin in vitro. Consistent with the overlapped expression pattern of FAM20C and Periostin, our data demonstrate for the first time that Periostin is a direct FAM20C-binding partner and that FAM20C phosphorylates Periostin in vitro.

## Introduction

Periodontium consists of root cementum, periodontal ligament (PDL), alveolar bone, gingiva and alveolar mucosa^1^. PDL is a soft, unmineralized connective tissue between the cementum and the alveolar bone, which functions to support teeth in their sockets as a shock absorber against mastication impact. PDL also functions as a sensory receptor and a cell reservoir for tissue homeostasis, repair and regeneration^1^. It is well known that fibroblast is the major cell type observed in PDL. PDL fibroblasts produce type I collagen, which is the major constituent of PDL extracellular matrices to support the ligament, while other non-collagenous proteins are found in the PDL region. However, the role(s) of these non-collagenous gene products for PDL tissue integrity and maintenance is still not fully clear.

Periostin was first identified as osteoblast-specific factor 2 (OSF-2) from mouse osteoblastic MC3T3-E1 cell cDNA library^2^, later renamed based on the expression patterns in periosteum and PDL^3^. Periostin is highly expressed in collagen-rich connective tissues subjected to constant mechanical loading in vivo and associated with various tissue homeostasis and pathology including bone, skin, PDL, cardiac valves, muscle injury, vascular injury, myocardial infarction, epithelial ovarian cancer, colorectal cancer, pulmonary vascular remodeling, bronchial asthma and oral cancer^4–6^. Periostin is a 90-kDa secretory protein that has several domains composed of an amino-terminal small cysteine-rich domain in the EMILIN family (EMI domain), tandem repeats of 4 Fasciclin (Fas) I domains (also called repeated domains (RDs)), and a carboxyl-terminal region (CTR)^7^. Periostin is reported to bind to several extracellular matrix (ECM) molecules and growth factors such as type I collagen and Notch1^8,9^. It has been reported that Periostin directly interacts with Fibronectin^10^ through its EMI domain, while Tenascin-C^7^, CCN3^11^ and BMP1^12^ through Fas I domain. These findings suggest that Periostin is a multi-functional protein possibly through orchestrating these binding proteins inside and outside of the cell^13^.

FAM20C is a member of “Family with sequence similarity 20”, consisting of three members; FAM20A, FAM20B and FAM20C. FAM20C, also known as DMP4 in mice^14^, is highly expressed in chondrocytes, osteoblasts, osteocytes, odontoblasts, ameloblasts and cementoblasts as well as dentin, enamel and bone matrices^15^. The expression pattern of FAM20C suggests that it has an important role in the formation of these mineralized tissues and subsequent mineralization process. Recently, FAM20C was identified as an intracellular kinase, namely golgi casein kinase (GCK), which was first described in lactating mammary glands that GCK enzymatically phosphorylates endogenous casein^16^. GCK/FAM20C phosphorylates secretory pathway proteins within Ser-X-Glu/phospho-Ser (SXE/pS) motif, which many of small integrin-binding ligand, N-linked glycoprotein (SIBLING) family members possess^17^. SIBLING proteins are known to function as nucleaters or inhibitors of biomineralization. It has been previously reported that acidic serine- and aspartate-rich motif (ASARM) peptide derived from osteopontin inhibits mineralization by binding to hydroxyapatite in a phosphorylation-dependent manner^18^. FAM20C is also known as a causative gene for Raine syndrome (OMIM#259775)^19^. Raine syndrome is an autosomal recessive rare disorder characterized by generalized osteosclerosis with periosteal bone formation, manifesting distinctive facial phenotype^20^. Oral/craniofacial phenotypes have been reported that the non-lethal type of Raine syndrome patients have small teeth with enamel dysplasia^20^, enlarged gingiva^21^ and amelogenesis imperfecta with significant gingivitis^22^. A previous report suggested that FAM20C is required for maintenance of healthy periodontal tissues^23^. As FAM20C functions as an intracellular protein kinase in the secretory pathway, it is reasonable to speculate that there might be some secretory proteins working together with FAM20C in PDL tissues.

Here we show that Periostin was identified during the course of FAM20C protein purification by Mass Spectrometry analysis. FAM20C interacted with Periostin in cell cultures. Using recombinant proteins, FAM20C directly bound to Periostin in vitro. We further narrowed down the binding domain of FAM20C and found that Fas I domain in Periostin is necessary for the binding to FAM20C. Immunohistochemical analysis demonstrated that immunolocalization of FAM20C was observed in murine PDL, which was overlapped with that of Periostin. Periostin was phosphorylated by FAM20C in vitro.

## Results

### Identification of Periostin during FAM20C protein purification process by Mass Spectrometry (MS) analysis

We intended to obtain FAM20C proteins, thus FAM20C-stablely transfected clones were first generated by transfecting FAM20C-WT-V5/His or FAM20C-D478A-V5/His expression vector into HEK 293 cells. HEK 293 cells were selected for their higher transfection efficiency.

**Figure 1:**
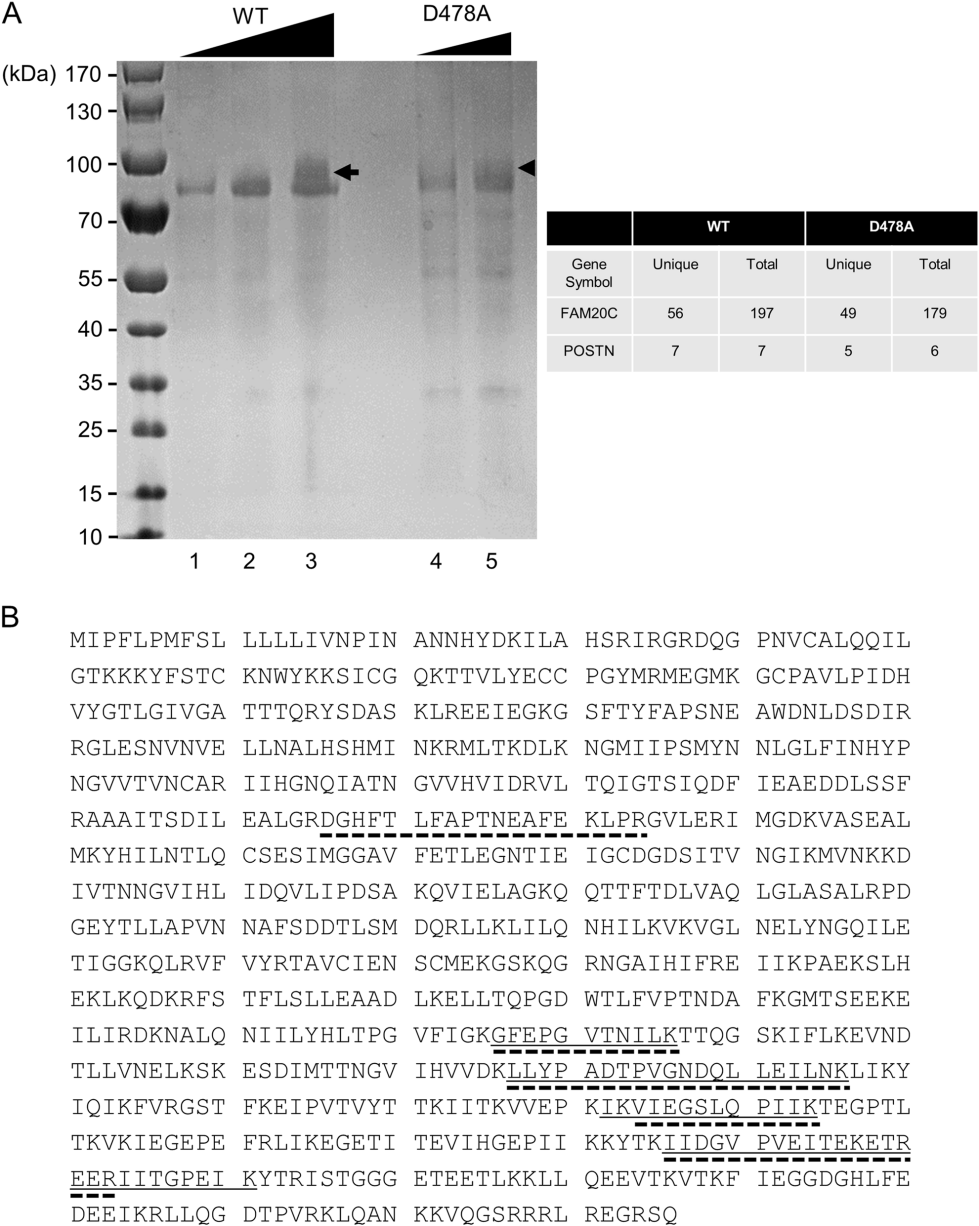
Identification of Periostin during FAM20C protein purification process. (**A**) Various doses of FAM20C WT-V5/His and FAM20C D478A-V5/His proteins, both purified from the conditioned media, were electrophoresed, stained with Coomassie Brilliant Blue (CBB; **A**, left) and the CBB-positive bands (lane 3, indicated by an arrow and lane 5 indicated by an arrowhead) were subjected to Mass Spectrometry analysis. The table on the right summarizes the numbers of unique peptides (unique) and total numbers of peptides (total) found by the analysis. For example, in case of FAM20C-WT, the analysis found a total of 197 peptides matching with FAM20C (from lane 3 sample indicated by an arrow) and among the 197 peptides, there are 56 unique peptides found. POSTN; Periostin. (**B**) Protein sequence of human Periostin isoform 1. The identified peptide sequences derived from FAM20C-WT-V5/His protein were indicated by underlines and those from FAM20C-D478A-V5/His were in dotted underlines.

The conditioned media from the FAM20C-WT-V5/His-transfected cell clone or the FAM20C-D478A-V5/His-transfected cell clone were collected, and FAM20C proteins were purified by Ni-NTA purification system. The expression of FAM20C-WT-V5/His and -D478A-V5/His were confirmed by Western blotting with both anti-V5 and anti-FAM20C antibodies after purification (data not shown). The purified FAM20C-WT-V5/His and FAM20C-D478A-V5/His proteins were prepared, electrophoresed, and the gel was stained with CBB. A single CBB-positive band was then found at a molecular weight corresponding to the size of FAM20C-WT-V5/His or -D478A-V5/His protein (~ 75 kDa band in Fig. 1). The purified FAM20C-V5/His fusion protein was detected at a slightly higher molecular weight position than previously reported^17^ likely due to the presence of V5/His tag (~ 5 kDa). Interestingly another band appeared at a higher molecular weight than the FAM20C-WT-V5/His or FAM20C-D478A-V5/His protein when the amount of FAM20C-WT-V5/His (Fig. 1A, lane 3, indicated by an arrow) or -D478A-V5/His (Fig. 1A, lane 5, indicated by an arrowhead) protein applied was increased, suggesting the presence of FAM20C-binding protein(s). These CBB positive bands with broader appearance were cut (Fig. 1A, indicated by an arrow and an arrowhead), further treated with trypsin and the digested peptides were subjected to protein identification analysis. To exclude the false-positive peptides, the data were analyzed with the filter setting condition (Xcorr2; 2.2, Xcorr3; 3.5) as previously reported^24^. This analysis revealed that the peptides of FAM20C-WT and -D478A were identified with a protein coverage of 58.73% (56 unique peptides and 197 total peptides found) and 59.25% (49 unique peptides and 179 total peptides found) (Fig. 1A, upper right table), respectively, indicating the successful purification of the proteins. Although the percentage of protein coverage was not high, we also identified the peptides of human endogenous Periostin (POSTN) likely derived from HEK293 cells. The Periostin peptides were found from both FAM20C-WT-V5/His and FAM20C-D478A-V5/His proteins (Fig. 1A, upper right table). The peptides identified by FAM20C-WT-V5/His contained 7 unique peptides of POSTN, i.e. residues 576–586 (GFEPGVTNILK), 627–646 (LLYPADTPVGNDQLLEILNK), 682–694 (IKVIEGSLQPIIK), 684–694 (VIEGSLQPIIK), 736–753 (IIDGVPVEITEKETREER), 736–750 (IIDGVPVEITEKETR), 754–761 (IITGPEIK), and those by FAM20C D478A-V5/His contained 5 unique peptides of POSTN, i.e. 266–284 (DGHFTLFAPTNEAFEKLPR), 576–586 (GFEPGVTNILK), 627–646 (LLYPADTPVGNDQLLEILNK), 684–694 (VIEGSLQPIIK), 736–753 (IIDGVPVEITEKETREER) (Fig. 1B).

### Binding of FAM20C to Periostin

**Figure 2:**
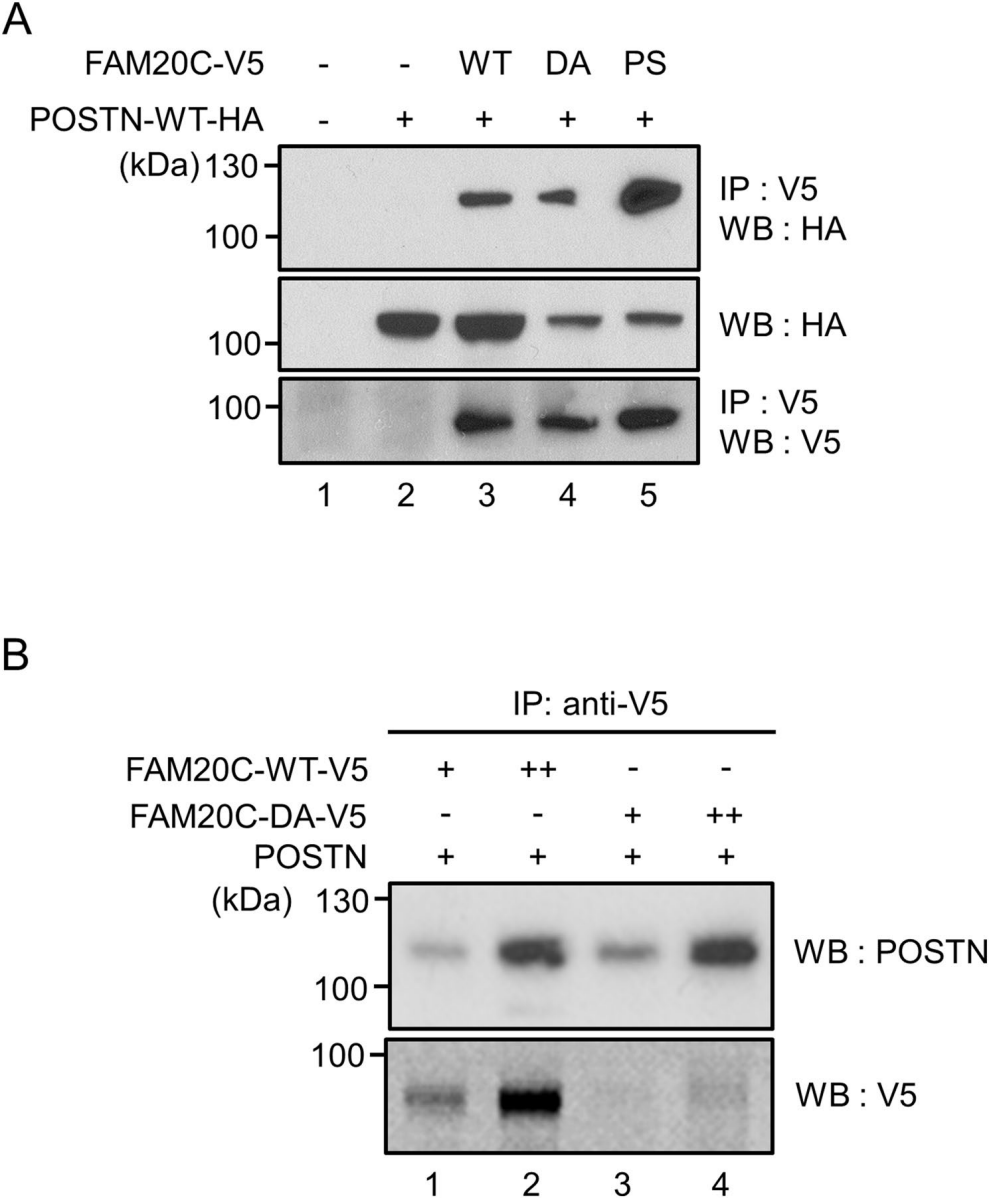
Binding of FAM20C to Periostin. (**A**) FAM20C binds to Periostin (POSTN) in cell cultures. HEK 293 cells were transiently transfected with POSTN-WT-HA and FAM20C-WT-V5/His, FAM20C-D478A-V5/His, or FAM20C P328S-V5/His. The cell lysates were collected, and immunoprecipitation (IP)-Western blot (WB) analyses were performed. The binding of POSTN to FAM20C-WT (WT, upper panel, lane 3), -D478A (DA, upper panel, lane 4), or -P328S (PS, upper panel, lane 5) was observed, indicating that this interaction did not require FAM20C kinase activity. (**B**) Direct binding of FAM20C to Periostin. FAM20C and POSTN proteins were incubated in vitro, immunoprecipitated with anti-V5 antibody, and subjected to WB analysis with anti-POSTN antibody. The direct binding was observed between FAM20C-WT-V5 and POSTN (upper panel, lanes 1 and 2) as well as FAM20C-DA-V5 and POST (upper panel, lanes 3 and 4) in a dose dependent manner.

To verify the interaction between FAM20C and POSTN, a kinase inactive form of FAM20C (D478A) and a Raine-syndrome mutation form (P328S) in addition to FAM20C-WT were used for the binding assay. HEK 293 cells were transiently transfected with POSTN-WT-HA and FAM20C-WT-V5/His, FAM20C-D478A-V5/His, or FAM20C-P328S-V5/His to investigate the interaction. The results demonstrated that POSTN-WT interacted with FAM20C-WT (Fig. 2A, upper panel, lane 3). POSTN-WT also interacted with both kinase inactive forms of FAM20C, i.e. D478A (Fig. 2A, upper panel, lane 4) and P328S (Fig. 2A, upper panel, lane 5), indicating that the interaction between FAM20C and POSTN does not require FAM20C’s kinase activity.

To determine whether the binding between POSTN and FAM20C was direct without the presence of other molecules, we examined the binding between recombinant POSTN protein produced by Sf21 baculovirus system and FAM20C-WT-V5/His or -D478A-V5/His protein purified from the conditioned media in a dose dependent manner. Proteins were incubated and the immunoprecipitation (IP)-Western blot analysis was performed. The results showed that the binding between POSTN and FAM20C-WT (Fig. 2B, upper panel, lanes 1 and 2) or FAM20C-D478A (Fig. 2B, upper panel, lanes 3 and 4) was clearly observed in a dose dependent manner. Our data demonstrate that FAM20C is a direct binding partner of Periostin.

### FAM20C binding domain locates in Fas I domain of Periostin

**Figure 3:**
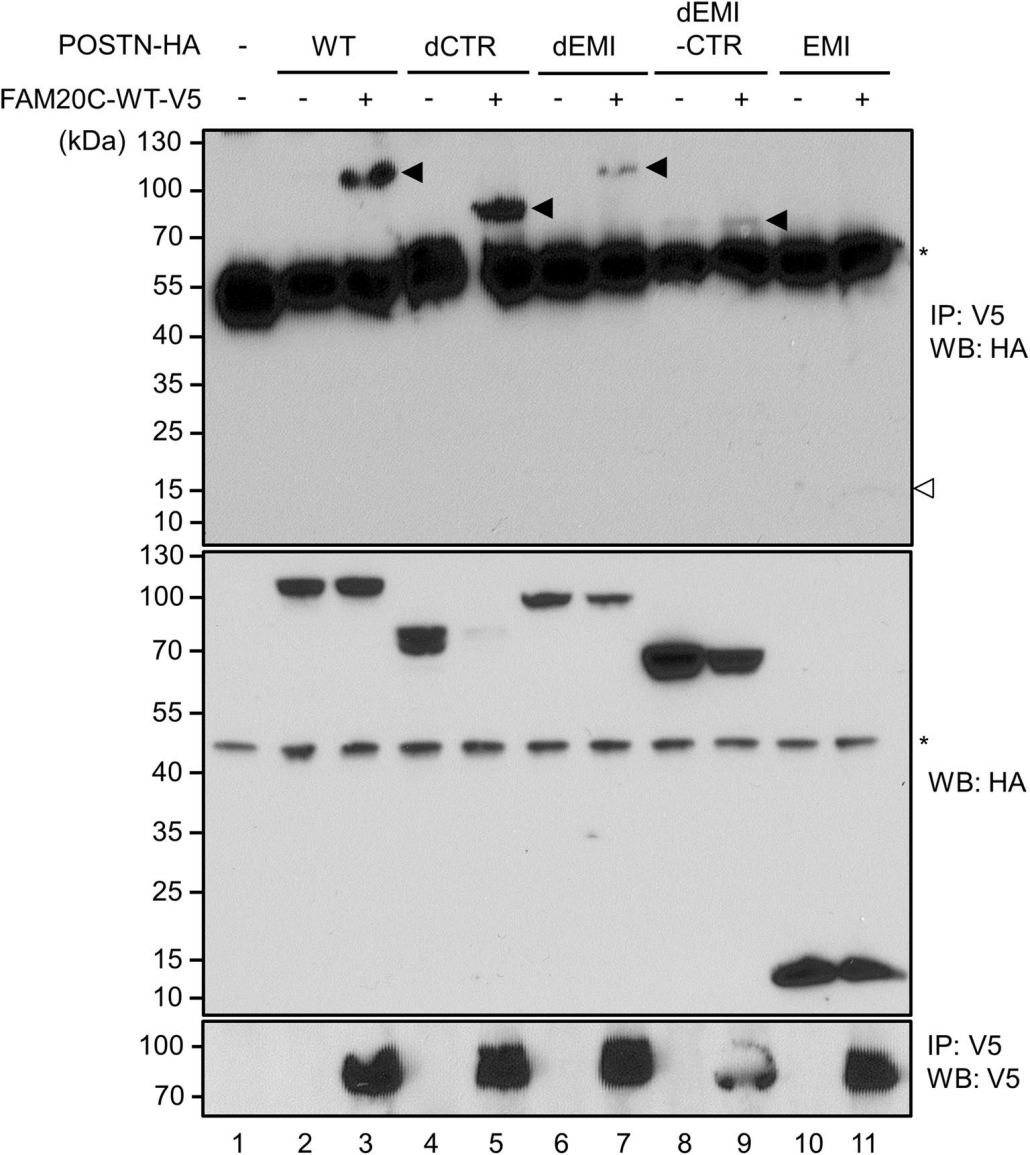
Fasciclin I domain in Periostin is the FAM20C-binding region. FAM20C-Periostin binding mapping. HEK 293 cells were transfected with POSTN-WT-HA, POSTN-dCTR-HA, POSTN-dEMI-HA, POSTN-dEMI-CTR-HA, or POSTN-EMI-HA and FAM20C-WT-V5/His. The binding between POSTN and FAM20C was investigated using cell lysates by immunoprecipitation (IP)—Western blotting (WB) analysis. The result showed that FAM20C interacted with POSTN-WT-HA (upper panel, lane 3, indicated by an arrowhead), POSTN-dCTR-HA (upper panel, lane 5, indicated by an arrowhead), POSTN-dEMI-HA (upper panel, lane 7, indicated by an arrowhead), and POSTN-dEMI-CTR-HA (upper panel, lane 9, indicated by an arrowhead), but not with POSTN-EMI-HA (upper panel, lane 11, indicated by an open arrowhead). The expression of all POSTN-HA constructs in the same cell lysate was confirmed by WB using anti-HA antibody (middle panel) and the level of immunoprecipitated FAM20C was confirmed by WB using anti-V5 antibody (lower panel). An asterisk indicates a non-specific band (upper and middle panels).

To further determine the region(s) within Periostin responsible for FAM20C binding, several deletion constructs of Periostin were used and the binding assay was performed in the similar manner as described above (Fig. 2A). HEK 293 cells were transiently transfected with FAM20C-WT-V5/His (FAM20C-WT-V5, indicated by +) and POSTN-WT-HA (WT), POSTN-dCTR-HA (dCTR), POSTN-dEMI-HA (dEMI), POSTN-dEMI-CTR-HA (dEMI-CTR) or POSTN-EMI-HA (EMI). The results demonstrated that immunoreactive bands to anti-HA antibody were shown when FAM20C-WT-V5 and forms of WT (Fig. 3, upper panel, lane 3), dCTR (Fig. 3, upper panel, lane 5), dEMI (Fig. 3, upper panel, lane 7), dEMI-CTR (Fig. 3, upper panel, lane 9) of POSTN were co-expressed. However, when FAM20C-WT and POSTN EMI were co-expressed, the binding was not observed (Fig. 3, upper panel, lane 11). Our results suggested that FAM20C binds to Fas I domain 1–4 (RD 1–4) of Periostin.

### Localization of FAM20C and Periostin in mouse periodontium tissues

To analyze the molecular distribution of FAM20C and Periostin in periodontal tissues, serial sections of mouse maxillary periodontium regions were stained using anti-FAM20C (a, b) or anti-Periostin (c, d) antibody. Non-immune goat immunoglobulin (IgG) was also used as a negative control for anti-FAM20C (e, f) and non-immune rabbit IgG for anti-Periostin (g, h). Distal root of maxillary first molar was selected as a region of interest. Panels (b, d, f, h) show higher magnification views of the open boxed areas in (a), (c), (e) or (g), respectively. Immunolocalization for FAM20C (brown color) was present in PDL (a, b), with preferential distribution at the bone surface region (b; arrows) and bone embedded Sharpey’s fibres (b; arrow heads). Periostin was present (brown color) in PDL (c, d) with strong positive signals along the thick PDL collagen, both at the bone surface and cementum surface regions of PDL (d; arrows), and bone embedded Sharpey’s fibres (d; arrow heads). No immunoreactivities were detected when non-immune IgG was used (e–h).

### FAM20C phosphorylates Periostin in vitro

We examined S-X-E FAM20C phosphorylation consensus sites in mouse Periostin and there were several potential phosphorylation sites (Fig. 5B). We then investigated whether Periostin protein could be phosphorylated by FAM20C in vitro. In vitro kinase assay was performed according to the previous reports^17,25^ and phosphorylated substances were enriched with Phos-tag agarose. Isolated phosphorylated proteins were separated by SDS-PAGE and detected by anti-Periostin antibody. Our results demonstrated that Periostin was detected when FAM20C WT (Fig. 5A, lane 4), but not D478A (Fig. 5A, lane 5), was incubated with Periostin. When Periostin alone was used in the absence of FAM20C protein, immunoreactivity to anti-Periostin antibody was not observed (Fig. 5A, lane 6). The data thus indicate that FAM20C phosphorylates Periostin in vitro.

## Discussion

There has been increasing evidence that Periostin plays an important role in periodontal tissue development. *Periostin* knock-out (KO) mice were generated and the homozygous mice showed periodontal tissue phenotypes^26–28^. In *Periostin* KO mice, PDL fibroblasts were irregularly distributed among collagen fibrils and collagen fibrils were disorganized^28^. While periodontium of WT and KO mice appeared intact when teeth were unerupted, after tooth eruption, dramatic periodontal defects were observed including enlarged gingival tissue, attachment loss, irregular PDL width, alveolar bone loss and external root resorption. Removal of masticatory forces partially rescued the PDL phenotype, suggesting the potential role of Periostin via transcription level^26^. It has also been reported that *Periostin* deficiency leads to decrease in collagen fibril diameter, which may explain why KO mice’s PDL collagen fibrils were susceptible to occlusal force and damaged^8^. Besides the PDL defect, *Periostin* KO mice exhibited dwarfism and enamel defects, suggesting that Periostin plays crucial roles in bone and tooth development^27^. Despite its possible critical role of Periostin reported in certain tissues including PDL, there has been so far no gene mutations found in humans.

**Figure 4:**
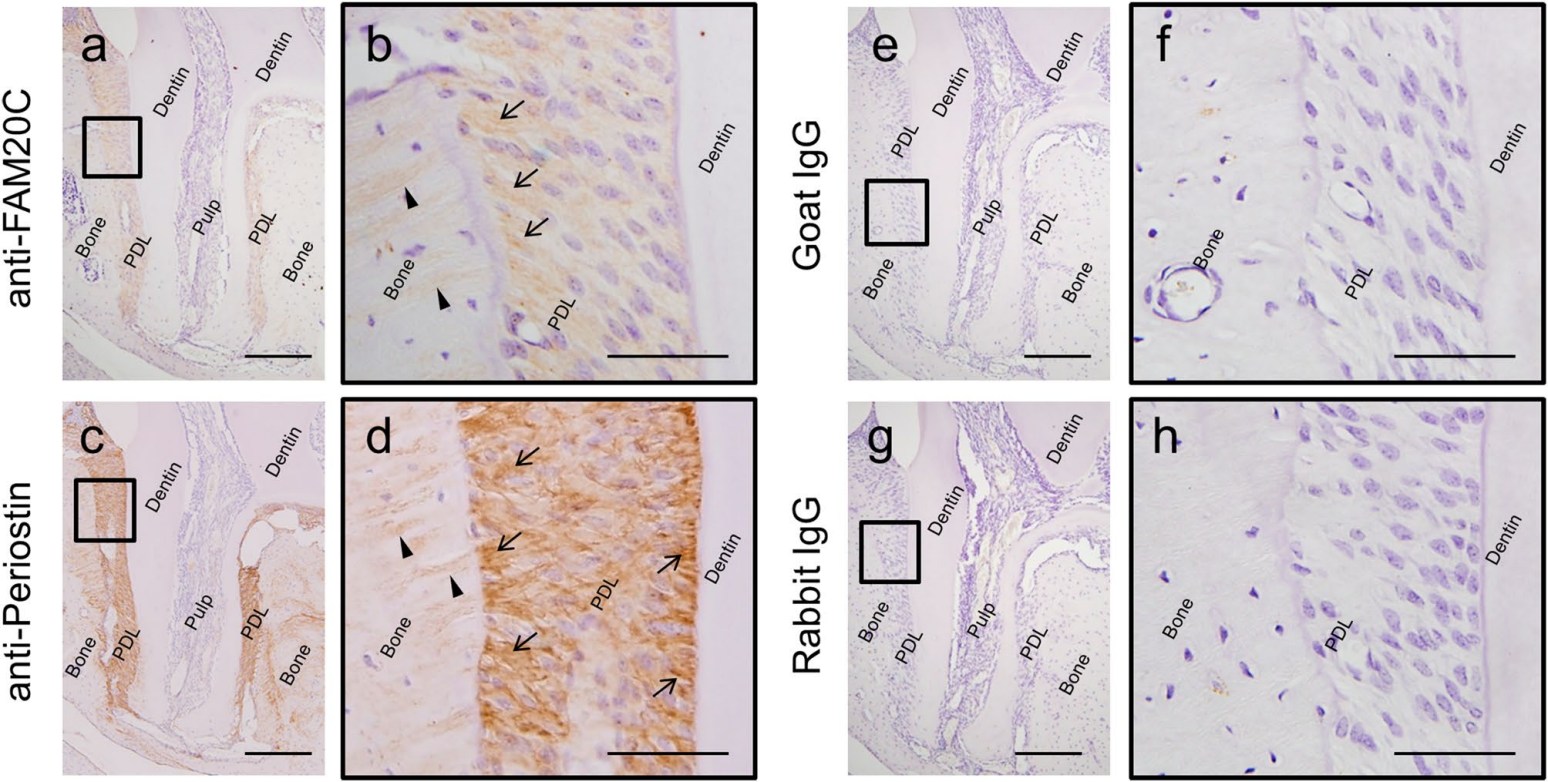
Immunohistochemical analyses of FAM20C and Periostin in mouse periodontal ligament tissues. Immunolocalization for FAM20C (brown color) was present in PDL (**a**, **b**), with preferential distribution at the bone surface region (b; arrows) and bone embedded Sharpey’s fibres (**b**; arrow heads). Periostin was present (brown color) in PDL (**c**, **d**) with strong positive signals along the thick PDL collagen, both at the bone surface and cementum surface regions of PDL (**d**; arrows), and bone embedded Sharpey’s fibres (**d**; arrow heads). No immunoreactivities were detected when non-immune goat immunoglobulin (IgG) was used as a negative control for anti-FAM20C (**e**, **f**), and non-immune rabbit IgG for anti-Periostin (**g**, **h**). Scale bar; 200 μm (**a**, **c**, **e**, **g**), 50 μm (**b**, **d**, **f**, **h**).

Among the FAM20 family, it has been reported that periodontitis may be a part of clinical phenotypic spectrum in FAM20A mutations^29^. Some reports showed the presence of periodontitis in non-lethal type of Raine syndrome, where FAM20C is mutated, but genotype–phenotype correlation was not established. Although FAM20A is considered to be an allosteric activator of FAM20C kinase^30^ and some clinical phenotypes in patients with FAM20A mutations are overlapped with those who with FAM20C mutations, it has not been ruled out that periodontitis in Raine syndrome patients is just coincidence. On the contrary, in mice, the involvement of FAM20C in periodontal tissues has been more extensively studied and established. The expression of *Fam20C* mRNA is detected in PDL fibroblasts at 5 and 7 weeks and FAM20C protein is expressed in PDL matrix in the incisor at 7 weeks^15^. Consistent with this, our data demonstrated that FAM20C is expressed in PDL tissues by immunohistochemistry (Fig. 4). More recently, conditional *Fam20C* KO mouse in cells expressing type I collagen (*Fam20C* cKO) was generated and showed periodontal disease phenotype. Since collagen fibers appeared thinner and unevenly distributed in these mice, this disrupts PDL structure, which likely allows direct infiltration of bacteria from periodontal pocket leading to periodontal disease. Additionally, in *Fam20C* cKO mice, the expression level of Periostin in PDL was dramatically reduced^23^. *Fam20C* global KO mouse (*Fam20C* −/− mouse) was generated by another group and demonstrated approximately 20% of mortality rate. Surviving *Fam20C* −/− mice showed decreased body weight and length, bone abnormalities and lack of tooth enamel^31^, manifesting similar clinical phenotypes to Raine syndrome patients. Therefore, these findings in mouse model studies and observations in human case reports highly suggest the possible relationship between FAM20C and Periostin in PDL, bone and enamel, and these two genes are genetically associated.

**Figure 5:**
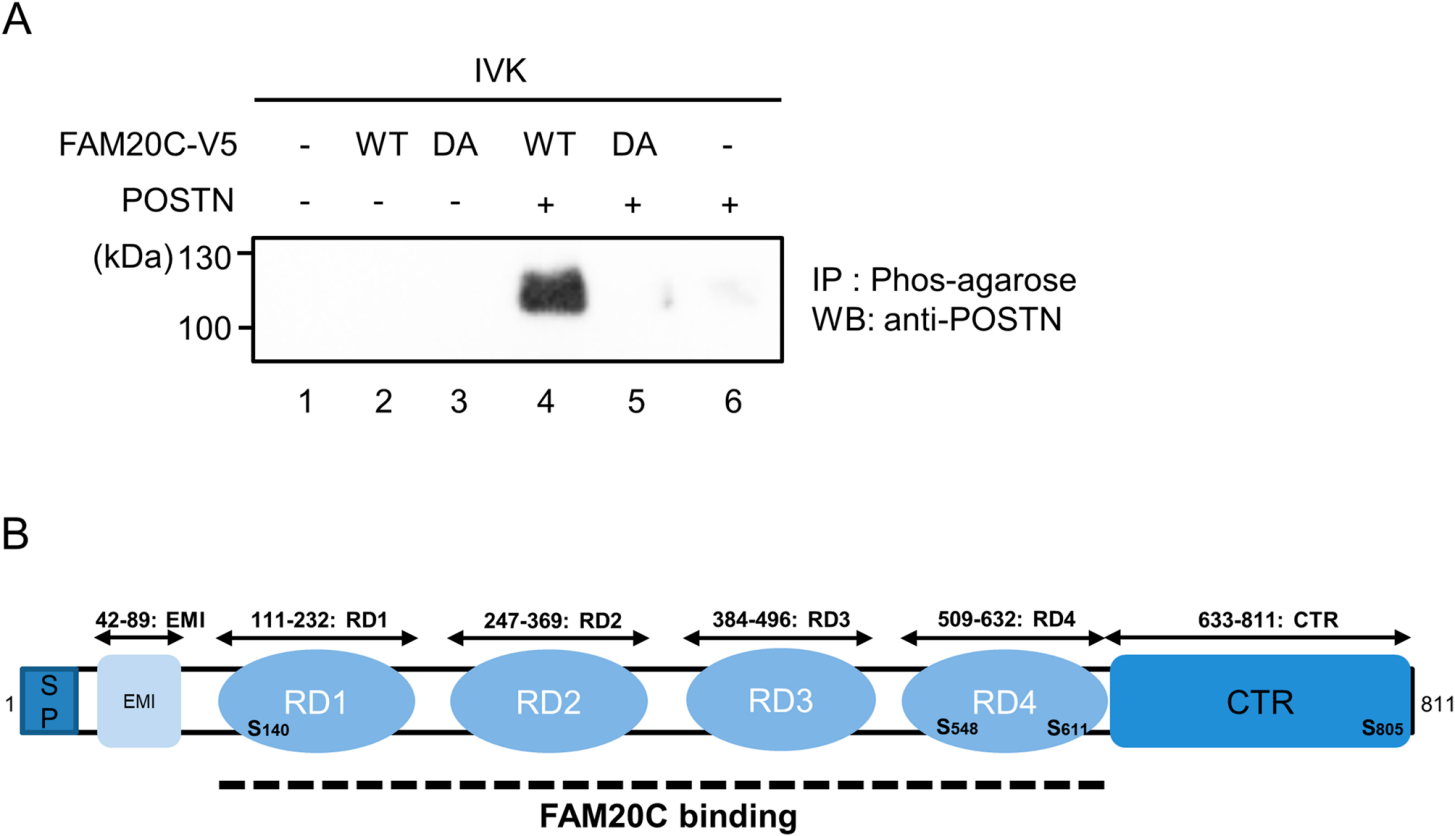
Periostin is phosphorylated by FAM20C WT, but not D478A kinase inactive form in vitro. (**A**) In vitro kinase assay was performed by incubating recombinant Periostin and FAM20C WT or kinase inactive D478A, Phos-tag agarose was added and phosphorylated proteins were isolated. Proteins were separated by 4–12% SDS-PAGE and Western blot analysis was performed using anti-Periostin antibody. Only when Periostin was incubated with FAM20C WT, but not D478, Periostin was detected, demonstrating that Periostin was phosphorylated by FAM20C-WT in vitro. (**B**) Schematic presentation of protein domain structures in mouse Periostin. Mouse Periostin protein structure is illustrated (811 amino acids based on NCBI Reference Sequence; NP_056599.1). Each domain was symbolized such as Signal peptide domain (SP), EMI domain (EMI), Fas I domains/repeated domains (RD1-4), and carboxyl-terminal region domain (CTR). A dotted line indicates FAM20C binding region based on our present study. Potential FAM20C phosphorylation Ser sites (S-X-E) are indicated in bold letters.

It has been known for almost 30 years that non-collagenous proteins in bone, dentin and enamel contain phosphoproteins with O-phosphoserine^32^. Despite the critical biological importance of phosphorylation in non-collagenous matrix proteins, kinase(s) responsible for phosphorylation have not been identified until recently. FAM20C kinase favors phosphorylation of an S-X-E/phosphorylated-S motif^17,25,33^. As illustrated in Fig. 5B, Periostin has several S-X-E motifs, showing one of potential candidate substrates for FAM20C kinase. Our data indicate that Periostin is phosphorylated by FAM20C (Fig. 5A), however, we could not identify the precise phosphorylation site(s) in Periostin using MS analysis due to lack of availability of the peptides containing these motifs (Fig. 1B). Four S-X-E motifs are found in mouse Periostin (S^140^-N-E, S^548^-E-E, S^611^-K-E, S^805^-R-E; based on NCBI Reference Sequence; NP_056599.1), of which three of them are conserved between mouse and human Periostin (S^138^-N-E, S^546^-E-E, S^609^-K-E; based on NCBI Reference Sequence; NP_006466.2). The conserved three S-X-E motifs are located within Fas I domains, i.e. S^140^-N-E in mouse RD1, S^548^-E-E and S^611^-K-E in mouse RD4. A possible reason why we could not retrieve any S-X-E motif peptides from our MS analysis is likely due to the detection limitation. As trypsin was used for peptide digestion and due to the presence of K or R residues near S-X-E motif, the length of peptide is too short to detect by the analysis. For example, S^546^-E-E containing peptide is supposed to be digested as “GMTSEEK”, and S^609^-K-E containing peptide as “SK”. At this point, it is still unclear why we could not detect any S^138^-N-E containing motif, however the peptide detection setting is normally 7–20 peptides and this S^138^-N-E containing peptide may be too long to detect. Further studies are required to verify the location of phosphorylation site(s) in Periostin.

The potential molecular function of Periostin has been characterized. It has been reported that Periostin is associated with cell proliferation, migration and activation of the survival signaling pathway PI3K/AKT/mTOR^34^. More recently, functional domain of this Periostin’s biological effect has been identified and reported by two independent groups; one is that a peptide sequence (amino acids 142–151) of Periostin stimulates chemotactic migration, adhesion, proliferation and endothelial tube formation of human endothelial colony forming cells in vitro^35^ and another that monoclonal antibodies that recognize amino acids 136–151 of Periostin inhibit Periostin-induced migration of human endothelial colony forming cells^36^. Interestingly, the functional domain reported in both studies is located within Fas I domain of Periostin, more specifically in RD1 which contains S^138^-N-E motif (Fig. 5B). Therefore, this suggests that phosphorylation by FAM20C may regulate Periostin-mediated cell functions and it is of our particular future interest to investigate the biological role of phosphorylated Periostin and its molecular function in both healthy periodontal tissues and periodontal disease.

## Materials and methods

### Ethics statement

The use of animals and all animal procedures in this study were approved by the Institutional Animal Care and Use Committee (IACUC) at Boston University Medical Campus (approved protocol number: AN-15050), and all efforts were made to minimize suffering animals. This study was performed in accordance with the NIH Guide for the Care and Use of Laboratory Animals.

### Cell culture

The human embryonic kidney (HEK) 293 cells were maintained as previously described^37,38^ and used in this study.

### Reagents and antibodies

X-tremeGENE 9 DNA transfection reagent was obtained from Roche Life Science. Recombinant mouse Periostin protein (2955-F2) was obtained from R&D Systems. The antibodies used in this study were as follows; anti-V5 (Life Technologies), anti-HA (clone 12CA5, Roche Life Science), anti-HA high affinity (clone 3F10, Roche Life Science), goat polyclonal anti-FAM20C (Santa Cruz Biotechnology), and rabbit polyclonal anti-Periostin (ab14041, Abcam) antibody. Rabbit polyclonal anti-Periostin antibody against RD1 domain of Periostin previously generated^39^ was used in the immunohistochemical analysis.

### FAM20C and Periostin expression vectors

Human FAM20C expression vector constructs, including wild-type (WT), Raine syndrome-mutant form (P328S), and a mutant form lacking its kinase activity (D478A) were generated by PCR methods as previously reported^40^. The plasmids harboring FAM20C-WT, FAM20C-D478A, and FAM20C-P328S cDNAs followed by V5—6XHis-tag (pcDNA3.1-FAM20C-WT-V5/His, pcDNA3.1-FAM20C-D478A-V5/His and pcDNA3.1-FAM20C-P328S-V5/His vectors) were used in this study. Mouse Periostin expression vectors previously generated in pCAGI-puro vector^7^ were used in this study.

### Purification of FAM20C proteins

The stable transfected HEK 293 cell clones that overexpress FAM20C-WT-V5/His or FAM20C-D478A-V5/His were generated and FAM20C-V5/His proteins were purified in the same manner as previously described^41^. Briefly, cells were transiently transfected using X-tremeGENE 9 DNA transfection reagent with pcDNA3.1-FAM20C-WT-V5/His and pcDNA3.1-FAM20C-D478A-V5/His according to the manufacturer’s protocol. The transfected cells were treated with 400 μg/ml of G418 neomycin analogue and further cultured. Ten of single colony-derived clones transfected with either FAM20C-WT or FAM20C-D478A were isolated, further cultured and the expression of FAM20C proteins was verified by Western blotting with anti-V5 antibody. The cell clone that expressed the strongest band intensity for FAM20C-WT-V5/His or FAM20C-D478A-V5/His was chosen, further cultured in a larger scale for protein production, and the conditioned media of FAM20C-WT-V5/His and FAM20C-D478A-V5/His were collected. The conditioned media were centrifuged at 1500 rpm for 5 min, the supernatant was incubated with Ni–NTA agarose beads (Qiagen), and FAM20C-WT-V5/His and FAM20C-D478A-V5/His proteins were purified as previously described^38, 41^. The purified proteins were dialyzed against distilled water, lyophilized and resuspended in distilled water. The protein concentration was measured and the purified proteins were kept at -20 °C until use.

### Protein identification by mass spectrometry (MS) analysis

Various amounts of purified FAM20C-WT-V5/His (2.5, 5.0 and 7.5 µg) and FAM20C-D478A-V5/His (2.5 and 5.0 µg) proteins were prepared, applied to 4–12% SDS-PAGE, and the gel was stained with Coomassie Brilliant Blue R-250 (CBB). The CBB staining positive, selected bands were excised and the protein identification was analyzed at Taplin Mass Spectrometry Facility (Harvard Medical School). Peptide sequences were determined by matching protein databases with the acquired fragmentation pattern.

### Binding of FAM20C to Periostin

HEK 293 cells were plated onto 6 well culture plates at a density of 3 × 10^5^ cells/well and transfected with mouse Periostin with C-terminal HA tag (pCAGI-puro delta Periostin (b-)-WT-HA; POSTN-WT-HA) and FAM20C-WT-V5/His, FAM20C-D478A-V5/His, or FAM20C-P328S-V5/His. Total amount of cDNA was kept constant by supplementation with empty vector. After 24 h of transfection, cell lysates were collected, immunoprecipitated with anti-V5 antibody and subjected to Western blotting (WB) analysis using anti-HA antibody to identify the binding. The same membrane was stripped by stripping buffer, and WB with anti-V5 antibody was performed to verify the expression of FAM20C-V5/His proteins. An aliquot of the same cell lysates was subjected to WB analysis with anti-HA antibody to verify the expression of POSTN-WT-HA.

### In vitro binding assay

The mouse recombinant Periostin and FAM20C-WT-V5/His or FAM20C-D478A-V5/His proteins were prepared in FAM20C protein’s dose dependent manner and incubated in PBS where the total amount of proteins per sample was kept constant by adding bovine serum albumin (BSA). Samples were then immunoprecipitated with anti-V5 antibody followed by Western blot (WB) analysis with anti-Periostin (Abcam) antibody. The same membrane was stripped, and WB with anti-V5 antibody was performed to verify the expression of FAM20C-V5/His proteins.

### Mapping of FAM20C-binding domain within Periostin

Various deletion mutant forms of pCAGI-puro delta Periostin (b-)-HA generated previously^7^ were used to identify the interaction domain of Periostin with FAM20C. The following was the deletion forms; Periostin (POSTN)-WT-HA, Periostin delta CTR-HA (POSTN-dCTR-HA) lacking its C-terminal region, Periostin delta EMI-HA (POSTN-dEMI-HA) lacking EMI domain, Periostin delta EMI-CTR-HA (POSTN-dEMI-CTR-HA) lacking both EMI and CTR domains, and Periostin EMI-HA (EMI domain only). HEK 293 cells were transiently transfected with POSTN-WT-HA, POSTN-dCTR-HA, POSTN-dEMI-HA, POSTN-dEMI-CTR-HA, or POSTN-EMI-HA and FAM20C-WT-V5/His. Total amount of cDNA was kept constant by supplementation with empty vector. After 24 h of transfection, cell lysates were collected and the binding domain was identified in the same manner as described above.

### Immunohistochemistry

The maxillary periodontal tissue including molars were dissected from mouse neonates (C57BL/6 strain, male, postnatal day 28), fixed, decalcified and embedded in paraffin. Immunohistochemical staining was performed as previously described^37^ with anti-FAM20C antibody (1:100 dilution), anti-Periostin antibody (1:400 dilution)^39^ or a non-immune goat or rabbit immunoglobulin (IgG) (at the same concentration as primary antibodies) as negative controls. The immuno-reactivity was amplified using VECTASTAIN Elite ABC HRP kit (Vector Laboratories Inc.). The sections were counterstained with hematoxylin (Sigma).

### In vitro kinase assay coupled with Phos-tag agarose and Western blot analysis

Kinase assays were performed in 20 μl reaction mixture containing wit 1 mM cold ATP (Thermo Fisher), 50 mM Tris–HCl pH 7.0, 10 mM MnCl_2_, 250 ng of recombinant POSTN and 500 ng of FAM20C-WT-V5/His or -D478A-V5/His protein. The total protein amount was kept constant supplemented by BSA. The mixtures were incubated for 30 min at 30 °C, Phos-tag agarose (Wako USA) was added, further incubated for 1 h at 4 °C, washed with lysis buffer and SDS sample buffer was added. Reaction products were separated by 4–12% SDS-PAGE and detected by Western blotting using anti-Periostin antibody.

## Supplementary information

Supplementary Information 1
