# Supplementary material for: FAM20C directly binds to and phosphorylates Periostin
Source: Sci Rep. 2020 Oct 13;10:17155. doi: 10.1038/s41598-020-74400-6 (PMC7555550; doi:10.1038/s41598-020-74400-6)

Supplementary Information

FAM20C directly binds to and phosphorylates Periostin

Ju-Hsien Lin, I-Ping, Lin, Yoshio Ohyama, Hanna Mochida, Akira Kudo, Masaru Kaku, and Yoshiyuki Mochida

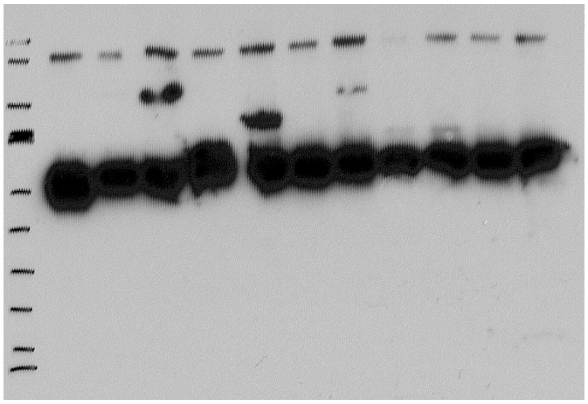
1 min exposure

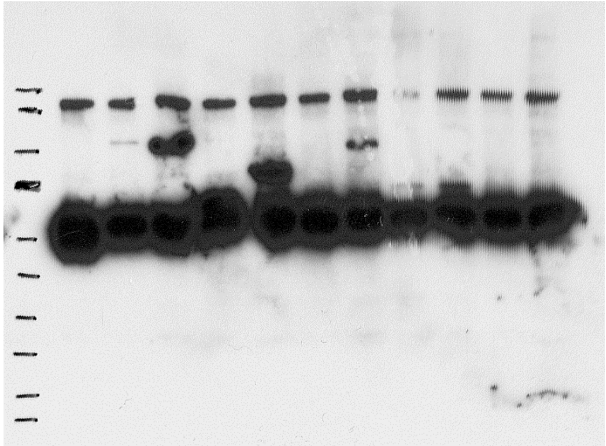
3 min exposure

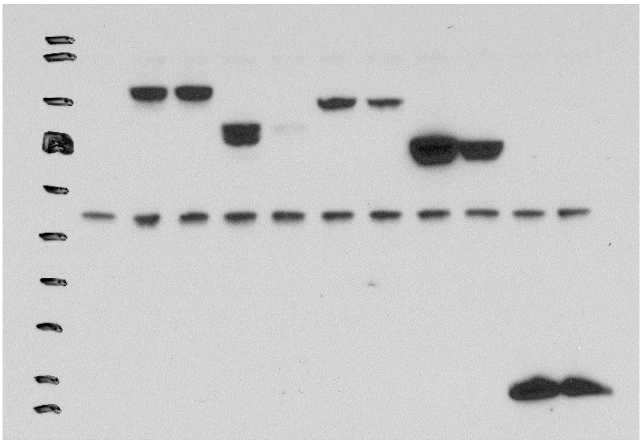

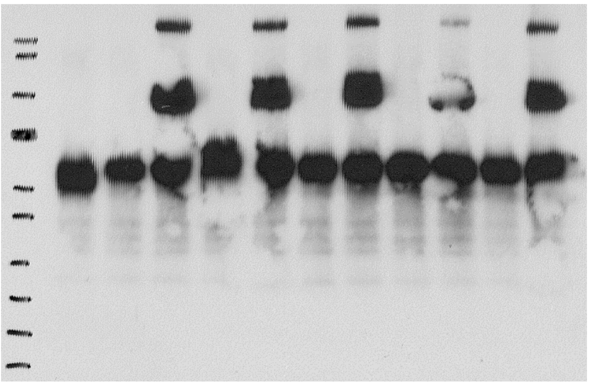

Supplement: Supplementary file 1 — Supplementary Information 1 [file 41598_2020_74400_MOESM1_ESM.pdf]
